# Effects of Maternal Cigarette Smoking on Pulmonary Function, Respiratory Muscle Strength, Functional Exercise Capacity, and Skeletal Muscle Oxygenation During the Second Trimester of Pregnancy

**DOI:** 10.3390/medicina62091743

**Published:** 2026-09-10

**Authors:** Ramazan Bülbül, Buğra Kerget, Ayşegül Evren Dilmaç, Zehra Deniz Zor Aksakal, Alperen Aksakal

**Affiliations:** 1 Department of Obstetrics and Gynecology, School of Medicine, Aksaray University, 68200 Aksaray, Turkeydrferhan68@hotmail.com; 2 Department of Pulmonary Diseases, School of Medicine, Aksaray University, 68200 Aksaray, Turkey; 3 Department of Obstetrics and Gynecology, Erzurum City Hospital, Yakutiye, 25240 Erzurum, Turkey; 4 Department of Pulmonary Diseases, School of Medicine, Ataturk University, Yakutiye, 25240 Erzurum, Turkey

**Keywords:** pregnancy, cigarette smoking, near-infrared spectroscopy, pulmonary function, respiratory muscle strength, six-minute walk test

## Abstract

*Background and Objectives***:** Cigarette smoking during pregnancy may adversely affect maternal respiratory function and exercise performance. This study aimed to compare pulmonary function, respiratory muscle strength, functional exercise capacity, and quadriceps muscle oxygenation between smoking and non-smoking women during the second trimester of pregnancy. *Materials and Methods***:** In this prospective cross-sectional study, 110 women in the second trimester of pregnancy (55 smokers and 55 non-smokers) underwent pulmonary function testing, respiratory muscle strength assessment, a six-minute walk test (6MWT), and quadriceps muscle oxygenation measurement using near-infrared spectroscopy (NIRS). *Results***:** Baseline demographic and obstetric characteristics were comparable between the groups (all *p* > 0.05). Compared with non-smokers, pregnant smokers had significantly lower forced vital capacity (FVC) and forced expiratory volume in one second (FEV1), including % predicted values and z-scores, as well as a lower FEV1/FVC ratio (all *p* < 0.001). Smokers also had lower maximal inspiratory pressure (MIP) and maximal expiratory pressure (MEP) (both *p* < 0.001) and a shorter six-minute walk distance (6MWD) (*p* < 0.001). In addition, smokers exhibited lower resting and end-exercise quadriceps muscle oxygen saturation (SmO_2_), together with a greater exercise-induced decline in SmO_2_ (ΔSmO_2_) (all *p* < 0.001). In multivariable regression analysis, pack-years (β = −0.512, *p* < 0.001) and gestational age (β = −0.217, *p* = 0.006) were independent predictors of ΔSmO_2_. *Conclusions***:** Maternal smoking during the second trimester is associated with modest reductions in pulmonary function, respiratory muscle weakness, reduced exercise capacity, and compromised skeletal muscle oxygenation. These findings suggest that the physiological effects of smoking extend beyond the lungs to peripheral muscle oxygen utilization and further emphasize the importance of early smoking cessation during pregnancy.

## 1. Introduction

Pregnancy is associated with physiological adaptations in the respiratory system that ensure adequate oxygen delivery to both the mother and the developing fetus [1]. Hormonal and mechanical changes alter respiratory mechanics, ventilation, and pulmonary function throughout gestation [2]. Although these adaptations are physiological, distinguishing them from pathological conditions affecting maternal and fetal health remains clinically important [3].

Cigarette smoking remains one of the most important modifiable risk factors during pregnancy despite widespread awareness of its harmful effects. Maternal smoking has been associated with impaired oxygen transport, endothelial dysfunction, chronic inflammation, and increased oxidative stress, all of which may compromise maternal exercise capacity and tissue oxygenation [4]. Furthermore, smoking during pregnancy has been linked to adverse obstetric and neonatal outcomes, including fetal growth restriction, preterm birth, placental complications, and low birth weight [5]. While these adverse effects are well recognized, their impact on maternal respiratory muscle performance, functional exercise capacity, and peripheral skeletal muscle oxygenation during pregnancy has not been adequately investigated [6].

Numerous studies have demonstrated the detrimental effects of maternal cigarette smoking on both maternal and fetal health. In addition to its adverse pregnancy outcomes, tobacco smoke exposure has been associated with increased oxidative stress, systemic inflammation, endothelial dysfunction, and reduced oxygen-carrying capacity, all of which may impair maternal respiratory function and tissue oxygen delivery [7,8]. Several studies have evaluated pulmonary function in pregnant smokers and reported significantly lower forced vital capacity (FVC), forced expiratory volume in one second (FEV_1_) and mid-expiratory flow rates compared with non-smoking pregnant women, suggesting that cigarette smoking negatively affects maternal lung function during gestation [9,10,11]. However, these studies have primarily focused on conventional spirometric parameters, and the potential effects of maternal smoking on respiratory muscle strength have received little attention. Although respiratory muscle strength has been investigated in healthy pregnancies, no study has specifically compared maximal inspiratory and expiratory pressures between smoking and non-smoking pregnant women.

Therefore, the present study aimed to compare pulmonary function, respiratory muscle strength, functional exercise capacity, and peripheral skeletal muscle oxygenation between smoking and non-smoking women during the second trimester of pregnancy. We hypothesized that maternal smoking would be associated with impaired pulmonary function, reduced respiratory muscle strength, diminished exercise capacity, and altered skeletal muscle oxygenation. The findings of this study may contribute to the limited evidence regarding the effects of maternal smoking on maternal functional and physiological performance during pregnancy.

## 2. Materials and Methods

### 2.1. Study Design and Participants

This prospective cross-sectional study was conducted between July 2026 and August 2026 at the Department of Pulmonary Diseases, Aksaray University Training and Research Hospital, and the Department of Pulmonary Diseases, Atatürk University Faculty of Medicine. Pregnant women attending routine antenatal outpatient visits at both centers during the second trimester were consecutively screened for eligibility. Recruitment was conducted simultaneously at both centers, allowing the required sample to be enrolled within the predefined study period. The same standardized assessment protocol and identical testing equipment were used at both centers to ensure consistency of data collection. The second trimester was selected to provide a relatively stable physiological period for respiratory and exercise assessments, while avoiding the prominent early-pregnancy symptoms and the greater mechanical and cardiopulmonary burden that typically develop later in gestation.

A total of 110 pregnant women were included and allocated into two groups according to their smoking status: 55 pregnant women who continued to smoke during pregnancy and 55 non-smoking pregnant women. The groups were matched as closely as possible for maternal age, gestational age, and body mass index (BMI). Demographic and obstetric characteristics, including maternal age, gestational age, height, body weight, body mass index, gravidity, parity, and hemoglobin concentration, were recorded. Smoking-related characteristics, including daily cigarette consumption, duration of smoking, cumulative smoking exposure (pack-years), and changes in cigarette consumption after conception, were also documented.

All participants underwent pulmonary function testing, respiratory muscle strength assessment, and the six-minute walk test with simultaneous quadriceps muscle oxygenation monitoring using near-infrared spectroscopy. All assessments were performed sequentially on the same day under standardized environmental conditions and in the same order for all participants: pulmonary function testing, respiratory muscle strength assessment, and finally the six-minute walk test with NIRS monitoring. Participants were instructed to avoid strenuous physical activity and caffeine-containing beverages for at least 2 h before testing. Pregnant women in the smoking group were also instructed to refrain from smoking for at least 2 h before the assessments to minimize the acute effects of cigarette smoking on cardiorespiratory and peripheral oxygenation measurements.

The study protocol was approved by the Atatürk University Clinical Research Ethics Committee, and written informed consent was obtained from all participants before enrollment. The study was conducted in accordance with the principles of the Declaration of Helsinki.

### 2.2. Inclusion and Exclusion Criteria

Pregnant women aged 18–40 years with a singleton pregnancy who were in the second trimester and were able to understand and perform the study procedures were considered eligible for inclusion. Participants in the smoking group were required to report active cigarette smoking during pregnancy, whereas those in the non-smoking group were required to report no current cigarette use and no regular secondhand smoke exposure at home or in the workplace.

Eligible participants were required to be between 18 and 40 years of age, have a singleton pregnancy in the second trimester, attend regular antenatal follow-up visits, and be able to perform pulmonary function testing, respiratory muscle strength measurements (MIP/MEP), the six-minute walk test, and near-infrared spectroscopy (NIRS) assessments.

Participants with known chronic respiratory diseases, including asthma, chronic obstructive pulmonary disease, bronchiectasis, interstitial lung disease, or active pulmonary infection, were excluded. Additional exclusion criteria included known cardiovascular disease, neuromuscular, musculoskeletal, or neurological disorders limiting exercise performance or walking ability, multiple pregnancy, high-risk pregnancy, active obstetric complications, uncontrolled hypertension, preeclampsia, placenta previa, significant vaginal bleeding, threatened preterm labor, severe anemia (hemoglobin < 8 g/dL)**,** use of tobacco products other than conventional cigarettes (including electronic cigarettes, heated tobacco products, or smokeless tobacco), and any maternal or fetal condition considered by the attending obstetrician to contraindicate exercise testing. Participants with technically unacceptable pulmonary function, respiratory muscle strength, six-minute walk test, or NIRS measurements that did not meet predefined quality criteria were excluded from the final analyses.

### 2.3. Maximum Inspiratory Pressure and Maximum Expiratory Pressure Measurement

Respiratory muscle strength was evaluated by measuring maximal inspiratory pressure (MIP) and maximal expiratory pressure (MEP) using the RP Check MIP & MEP & SNIP device (MD Diagnostics Ltd., Chatham, UK). Before each testing session, the device was calibrated according to the manufacturer’s recommendations. Although device-specific reproducibility data in pregnant populations are limited, MIP and MEP have been previously used to assess respiratory muscle strength during pregnancy [6]. To improve measurement reliability, standardized positioning and instructions were used, at least three maneuvers were performed, and the highest reproducible value was recorded.

All measurements were performed with participants seated comfortably in an upright position while wearing a nose clip to eliminate nasal air leakage. For MIP assessment, participants were instructed to exhale completely to residual volume and then generate a maximal inspiratory effort against an occluded mouthpiece. For MEP assessment, participants first inhaled to total lung capacity and subsequently performed a maximal expiratory effort against the occluded mouthpiece. Each maneuver was repeated at least three times, and the highest reproducible value was recorded for analysis. A minimum rest period of one minute was allowed between successive attempts to minimize fatigue. Standardized verbal encouragement was provided throughout the testing procedure to ensure maximal participant effort. All measurements were performed in accordance with the recommendations of the American Thoracic Society (ATS) and the European Respiratory Society (ERS) for respiratory muscle strength assessment [12].

### 2.4. Pulmonary Function Testing

Pulmonary function testing was performed using a SpiroLab III spirometer (MIR Medical International Research, Rome, Italy). Before testing, all participants received standardized instructions regarding the preparation for spirometry in accordance with the 2019 American Thoracic Society/European Respiratory Society (ATS/ERS) technical standards [13]. The spirometry procedure was explained and demonstrated by experienced respiratory technicians at each center, using the same standardized testing protocol and identical spirometry equipment.

Participants performed forced expiratory maneuvers while seated and wearing a nose clip. A minimum of three acceptable forced expiratory maneuvers was obtained from each participant. Only tests fulfilling the ATS/ERS 2019 criteria for acceptability and repeatability were included in the analysis [13]. The highest values of FVC and FEV_1_ from acceptable maneuvers were recorded for statistical evaluation. Predicted values and lower limits of normal were automatically calculated by the spirometry software using the reference equations incorporated into the device. All pulmonary function tests were performed using identical equipment and standardized testing procedures at both centers to minimize measurement variability.

### 2.5. Near-Infrared Spectroscopy Assessment

Skeletal muscle oxygenation was continuously assessed using a portable NIRS device (MOXY Muscle Oxygen Monitor, Fortiori Design LLC, Hutchinson, MN, USA). The NIRS sensor was positioned over the belly of the right vastus lateralis muscle after the skin had been cleaned. The probe was securely fixed with adhesive tape and covered with an opaque dressing to minimize movement artifacts and interference from ambient light throughout the test.

Following sensor placement, participants rested briefly to obtain a stable baseline signal before the six-minute walk test (6MWT). Continuous muscle oxygenation recording was initiated immediately before the start of the 6MWT and continued uninterrupted until completion of the test.

The MOXY monitor provides real-time measurements of skeletal muscle oxygen saturation (SmO_2_), expressed as the percentage of oxygenated hemoglobin relative to total hemoglobin within the interrogated muscle tissue. All recorded data were stored in the device’s internal memory as comma-separated value (.csv) files and subsequently transferred to a Microsoft Excel database via Bluetooth for offline analysis.

For the present study, resting SmO_2_ immediately before the 6MWT and end-exercise SmO_2_ recorded at the completion of the test were included in the primary analyses. The change in muscle oxygen saturation during exercise (ΔSmO_2_) was calculated as the difference between resting and end-exercise SmO_2_ values.

### 2.6. Outcome Measures

The primary outcome of the study was the difference in SmO_2_ measured by NIRS between pregnant smokers and non-smokers during the six-minute walk test. Muscle oxygenation was evaluated using resting SmO_2_, end-exercise SmO_2_, and the exercise-induced ΔSmO_2_, calculated as the difference between resting and end-exercise values.

Secondary outcome measures included pulmonary function parameters, namely FVC, FEV_1_, FEV_1_/FVC ratio, peak expiratory flow (PEF), and forced expiratory flow between 25% and 75% of FVC (FEF_25–75_). Respiratory muscle strength was assessed using MIP and MEP. Functional exercise capacity was evaluated by the 6MWD, together with pre- and post-exercise heart rate, peripheral oxygen saturation (SpO_2_), and modified Borg dyspnea and leg fatigue scores. Demographic and obstetric characteristics, including maternal age, gestational week, body mass index, gravidity, parity, hemoglobin level, and smoking-related variables, were also recorded for descriptive analyses and adjustment in multivariable models.

### 2.7. Statistical Analysis

Statistical analyses were performed using IBM SPSS Statistics for Windows, Version 27.0 (IBM Corp., Armonk, NY, USA). The distribution of continuous variables was assessed using the Shapiro–Wilk test together with visual inspection of histograms and Q–Q plots. As most continuous variables were not normally distributed, data are presented as median (interquartile range [IQR]), whereas categorical variables are presented as frequencies and percentages. Comparisons between smoking and non-smoking pregnant women were performed using the Mann–Whitney U test for continuous variables and the chi-square test or Fisher’s exact test, as appropriate, for categorical variables. All statistical tests were two-sided, and a *p* value < 0.05 was considered statistically significant. No formal adjustment for multiple comparisons was applied to the secondary outcomes; therefore, these analyses were considered exploratory and interpreted with caution.

To identify independent factors associated with the exercise-induced change in skeletal muscle oxygen saturation (ΔSmO_2_), a multiple linear regression analysis was performed. Based on their clinical relevance and potential physiological influence on muscle oxygenation, pack-years of smoking, body mass index (BMI), gestational age, maximal inspiratory pressure (MIP), and hemoglobin concentration were entered simultaneously into the regression model. Linearity and homoscedasticity were assessed by visual inspection of residual plots, while the normality of residuals was evaluated using a histogram and normal P–P plot of standardized residuals. Multicollinearity was evaluated using tolerance and variance inflation factor (VIF) values. A tolerance value > 0.10 and a VIF < 5.0 were considered indicative of the absence of significant multicollinearity. The highest VIF was 4.63 for MIP; as all VIF values remained below the predefined threshold of 5.0, no variable was excluded from the model because of multicollinearity.

## 3. Results

The median maternal age was similar between the groups, being 34 (31.5–37.0) years in non-smokers and 34 (31.0–37.0) years in smokers. Baseline demographic and obstetric characteristics are presented in Table 1. There were no statistically significant differences between the groups with respect to maternal age, gestational age, height, body weight, body mass index, gravidity, parity, or hemoglobin concentration (all *p* > 0.05).

Pulmonary function test results are presented in Table 2. Compared with non-smoking pregnant women, those who smoked had significantly lower FVC and FEV_1_ values expressed as both % predicted and z-scores (all *p* < 0.001), whereas absolute FVC was also lower (*p* = 0.002) and absolute FEV_1_ was markedly reduced (*p* < 0.001). In addition, FEV_1_/FVC ratio and its corresponding z-score were significantly lower in the smoking group (both *p* < 0.001). Although absolute PEF and FEF_25–75_ values were comparable between the groups (*p* = 0.216 and *p* = 0.083, respectively), their % predicted values and z-scores were significantly lower in pregnant smokers (PEF % predicted, *p* = 0.004; PEF z-score, *p* = 0.003; FEF_25–75_% predicted, *p* = 0.015; FEF_25–75_ z-score, *p* = 0.016).

**Figure 1:**
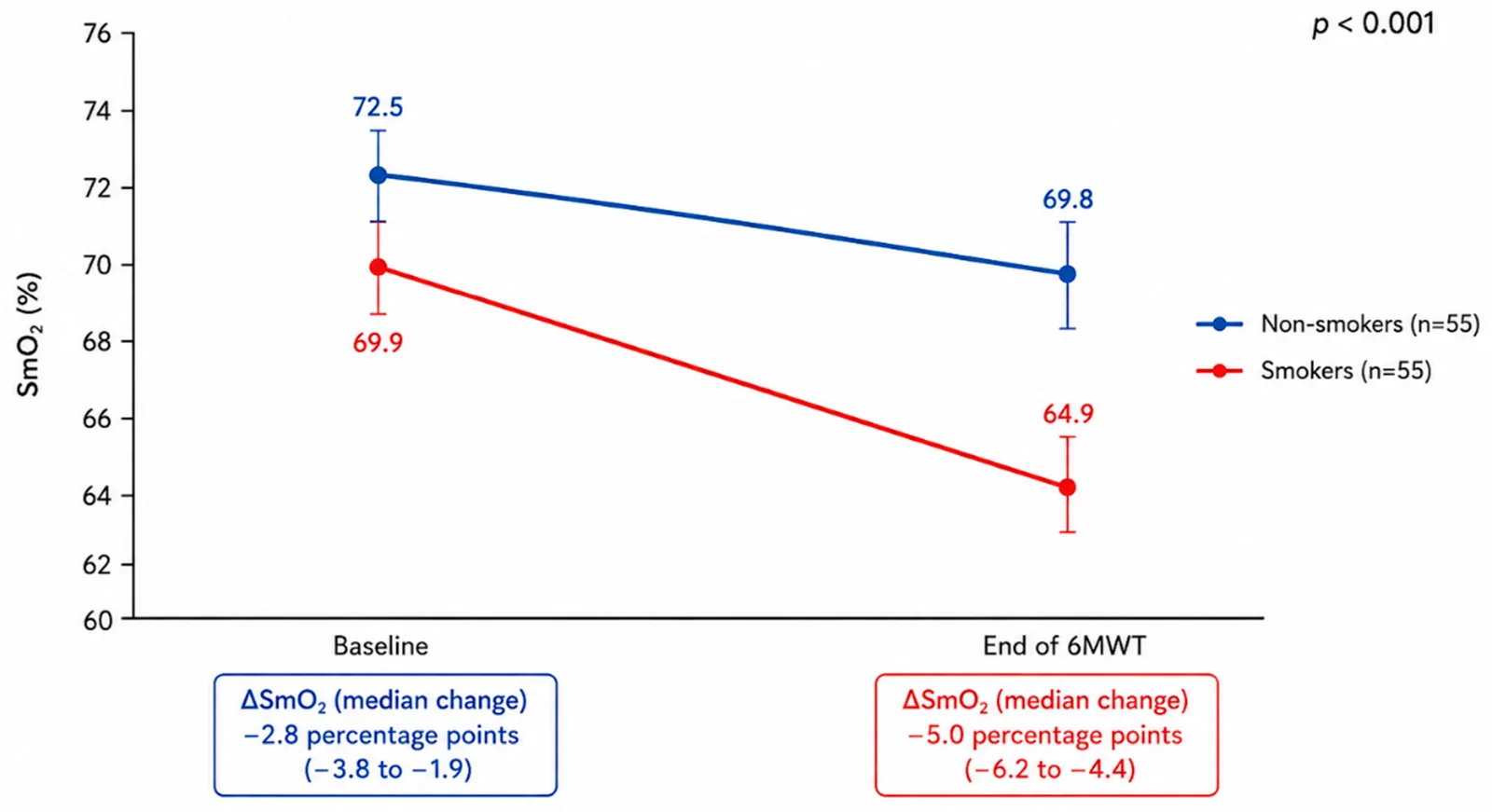
Changes in skeletal muscle oxygen saturation during the six-minute walk test in smoking and non-smoking pregnant women. Median skeletal muscle oxygen saturation (SmO_2_) measured by near-infrared spectroscopy (MOXY^®^) at baseline and immediately after the six-minute walk test (6MWT) in smoking and non-smoking pregnant women. Error bars represent the interquartile range (25th–75th percentile). Smoking pregnant women exhibited lower baseline and post-exercise SmO_2_ values and a greater decline in skeletal muscle oxygen saturation during the 6MWT compared with non-smoking pregnant women (*p* < 0.001).

Respiratory muscle strength, functional exercise capacity, dyspnea scores, and skeletal muscle oxygenation findings are presented in Table 3. Pregnant smokers demonstrated significantly lower MIP and MEP values, both as absolute measurements and as percentages of predicted values (all *p* < 0.001). Functional exercise capacity was also significantly reduced in the smoking group, as evidenced by a shorter 6-min walk distance (*p* < 0.001). Furthermore, smokers exhibited significantly lower baseline and end-exercise skeletal muscle oxygen saturation (SmO_2_), together with a significantly greater exercise-induced decline in SmO_2_ (ΔSmO_2_) compared with non-smokers (all *p* < 0.001) (Figure 1). While baseline Borg dyspnea scores were comparable between the groups (*p* = 0.684), post-exercise Borg dyspnea scores were significantly higher in pregnant smokers (*p* < 0.001).

The results of the multivariable linear regression analysis for factors associated with ΔSmO_2_ are presented in Table 4. After adjustment for BMI, gestational age, MIP, and hemoglobin concentration, pack-years of smoking (standardized β = −0.512, *p* < 0.001) and gestational age (standardized β = −0.217, *p* = 0.006) remained independently associated with ΔSmO_2_. In contrast, BMI, MIP, and hemoglobin concentration were not significant independent predictors (all *p* > 0.05). The final regression model explained 54.1% of the variance in ΔSmO_2_ (R^2^ = 0.541; adjusted R^2^ = 0.519; *p* < 0.001).

## 4. Discussion

The present study demonstrated that cigarette smoking during the second trimester of pregnancy was associated with modest reductions in pulmonary function, reduced respiratory muscle strength, lower functional exercise capacity, and altered peripheral skeletal muscle oxygenation. Pregnant smokers exhibited significantly lower FVC, FEV_1_, and FEV_1_/FVC values, together with lower predicted percentages and z-scores for PEF and FEF_25–75_, indicating modest but statistically significant differences in pulmonary function between the groups. Importantly, these differences should not be interpreted as evidence of clinically overt ventilatory impairment, as the spirometric values generally remained within expected physiological ranges. In addition, inspiratory and expiratory respiratory muscle strength was significantly lower in smokers, accompanied by a shorter six-minute walk distance. Furthermore, pregnant smokers had lower quadriceps muscle oxygen saturation both at rest and immediately after exercise, as well as a greater exercise-induced decline in muscle oxygenation, indicating impaired peripheral oxygen utilization during physical activity. The main novelty of the present study lies in the concurrent assessment of respiratory muscle strength, functional exercise capacity, and skeletal muscle oxygenation, which have been insufficiently investigated in pregnant smokers.

Pregnancy is accompanied by profound physiological adaptations in the respiratory system that support the increasing metabolic and oxygen demands of both the mother and the developing fetus [14]. Hormonal changes, particularly elevated progesterone levels, stimulate ventilation and contribute to an increase in tidal volume and minute ventilation, whereas the enlarging uterus progressively elevates the diaphragm and reduces functional residual capacity [1]. Despite these mechanical changes, spirometric indices generally remain within the normal range throughout uncomplicated pregnancy, and respiratory muscle strength is largely preserved owing to compensatory adaptations of the respiratory muscles [1]. Functional exercise capacity is also maintained in healthy pregnancies despite the increased cardiopulmonary workload associated with advancing gestation [15].

Cigarette smoking may adversely affect maternal respiratory physiology through multiple mechanisms, including chronic airway inflammation, oxidative stress, endothelial dysfunction, and impaired oxygen transport [1,14]. Previous studies have demonstrated that pregnant smokers exhibit lower spirometric parameters than non-smokers, particularly in measures reflecting expiratory airflow and small airway function [10,11]. In contrast, evidence regarding the effects of maternal smoking on respiratory muscle strength during pregnancy is currently lacking. Lemos et al. evaluated respiratory muscle strength in healthy primigravidae and reported that maximal inspiratory and expiratory pressures were preserved throughout pregnancy, with no significant association between gestational age and either MIP or MEP and no differences across pregnancy trimesters [6]. To our knowledge, however, no previous study has compared respiratory muscle strength between smoking and non-smoking pregnant women. Likewise, studies investigating skeletal muscle oxygenation using near-infrared spectroscopy in pregnant smokers are currently unavailable. Nevertheless, studies conducted in non-pregnant populations have shown that cigarette smoking is associated with impaired peripheral muscle oxygenation during exercise, suggesting that smoking-related vascular and metabolic alterations extend beyond the respiratory system [16].

Consistent with previous studies, pregnant smokers showed modest but statistically significant reductions in FVC, FEV_1_, and FEV_1_/FVC compared with non-smoking pregnant women, although these values remained within the expected physiological range. In addition, inspiratory and expiratory respiratory muscle strength was significantly reduced in the smoking group. Cigarette smoking may impair respiratory muscle performance through multiple mechanisms, including chronic oxidative stress, systemic inflammation, endothelial dysfunction, reduced mitochondrial efficiency, and chronic carbon monoxide exposure, all of which may compromise oxygen delivery and energy production within respiratory muscles [17,18,19]. These mechanisms may contribute to the lower inspiratory and expiratory muscle strength observed in pregnant smokers. The coexistence of respiratory muscle weakness and airflow limitation may have contributed to the reduction in vital capacity observed in these participants. Likewise, impaired pulmonary function together with reduced respiratory muscle strength may partly explain the lower functional exercise capacity, reflected by the shorter six-minute walk distance in pregnant smokers.

An important finding of the present study was that quadriceps muscle oxygen saturation was significantly lower in pregnant smokers both at rest and immediately after exercise, with a greater exercise-induced decline in SmO_2_ during the 6MWT. Despite comparable peripheral oxygen saturation measured by pulse oximetry before and after exercise, skeletal muscle oxygenation remained significantly impaired in the smoking group. This discrepancy suggests that systemic oxygen saturation may not fully reflect local skeletal muscle oxygenation. Although microvascular function and muscle metabolism were not directly assessed in the present study, the lower SmO_2_ observed in smokers may reflect several mechanisms, including altered local perfusion, mitochondrial dysfunction, and impaired muscle oxygen utilization. The greater decline in muscle oxygenation was also accompanied by higher post-exercise Borg dyspnea scores in pregnant smokers. Furthermore, multivariable regression analysis demonstrated that cumulative smoking exposure (pack-years) and gestational age were independent predictors of ΔSmO_2_, indicating that greater self-reported cumulative smoking exposure and advancing gestation were associated with a greater reduction in skeletal muscle oxygenation during exercise.

This study has several limitations. First, the cross-sectional design precludes causal inference and assessment of within-subject changes across pregnancy, while the inclusion of only second-trimester pregnancies may limit generalizability to other gestational periods. Physical activity, dietary habits, and socioeconomic status were not systematically assessed and may therefore represent residual confounders. Second, smoking status was based on self-report and was not biochemically confirmed using cotinine or exhaled carbon monoxide measurements. Although smokers refrained from smoking for at least 2 h before testing, acute effects of nicotine and carbon monoxide cannot be completely excluded. Third, multiple secondary outcomes were evaluated without formal adjustment for multiple comparisons, increasing the possibility of Type I error. The relatively high VIF for MIP (4.63) also indicates some degree of collinearity within the regression model and warrants cautious interpretation of its individual coefficient. Finally, skeletal muscle oxygenation was evaluated only at baseline and immediately after the six-minute walk test, without assessment of recovery kinetics. Despite these limitations, the standardized protocol and combined assessment of respiratory muscle strength, exercise capacity, and skeletal muscle oxygenation represent important strengths of the study.

## 5. Conclusions

In conclusion, cigarette smoking during the second trimester of pregnancy was associated with modest reductions in pulmonary function, although spirometric values remained within the expected physiological range, reduced respiratory muscle strength, lowered functional exercise capacity, and decreased quadriceps muscle oxygenation. Furthermore, cumulative smoking exposure was independently associated with a greater exercise-induced decline in skeletal muscle oxygenation. These findings suggest that the physiological effects associated with smoking during pregnancy extend beyond pulmonary function and may also involve peripheral muscle oxygen utilization. Early smoking cessation during pregnancy may therefore help preserve maternal respiratory and functional performance.

## Figures and Tables

**Table 1:** Demographic characteristics of the study groups.

Variable	Non-Smokers (*n* = 55)	Smokers (*n* = 55)	*p* Value
Age, years	33 (30–36)	34 (30–38)	0.484
Gestational age, weeks	21.5 (17.8–24.0)	21.1 (17.2–24.5)	0.830
Height, cm	164.5 (159.3–169.3)	165.9 (160.3–169.0)	0.425
Weight, kg	73.4 (64.1–77.6)	75.1 (65.8–79.3)	0.340
BMI, kg/m^2^	26.9 (23.8–28.8)	27.1 (25.1–29.1)	0.733
Hemoglobin, g/dL	11.7 (11.4–12.2)	11.9 (11.3–12.3)	0.749
Smoking exposure, pack-years *	0 (0–0)	14 (12–17)	—
Gravidity, *n*	2 (1–3)	2 (1–3)	0.798
Parity, *n*	1 (0–1)	1 (0–1)	0.777

* Data are presented as median (25th–75th percentile). Comparisons between groups were performed using the Mann–Whitney U test. BMI, body mass index. Pack-years are presented descriptively because this variable defines smoking exposure and is not applicable to the non-smoking group.

**Table 2:** Pulmonary function test results of the study groups.

Variable	Non-Smokers (*n* = 55)	Smokers (*n* = 55)	*p* Value
FVC, L	3.63 (3.43–3.83)	3.47 (3.30–3.64)	0.002
FVC, % predicted	102.1 (101.4–102.5)	96.8 (95.1–98.4)	<0.001
FVC, z-score	0.17 (0.12–0.21)	−0.27 (−0.41 to −0.13)	<0.001
FEV_1_, L	2.95 (2.82–3.15)	2.80 (2.66–2.94)	<0.001
FEV_1_, % predicted	102.1 (101.6–103.0)	92.6 (90.9–94.9)	<0.001
FEV_1_, z-score	0.17 (0.13–0.25)	−0.62 (−0.76 to −0.42)	<0.001
FEV_1_/FVC, %	81.9 (81.4–82.4)	80.9 (79.7–81.5)	<0.001
FEV_1_/FVC, z-score	−0.02 (−0.15 to 0.10)	−0.27 (−0.57 to −0.12)	<0.001
PEF, L/s	6.26 (5.96–6.64)	6.23 (5.90–6.39)	0.216
PEF, % predicted	100.82 (97.45–102.45)	98.23 (93.98–100.89)	0.004
PEF, z-score	0.07 (−0.21 to 0.20)	−0.15 (−0.50 to 0.07)	0.003
FEF_25–75_, L/s	3.53 (3.26–3.72)	3.44 (3.26–3.57)	0.083
FEF_25–75_, % predicted	98.73 (94.37–104.49)	95.25 (92.77–100.45)	0.015
FEF_25–75_, z-score	−0.08 (−0.38 to 0.30)	−0.32 (−0.48 to 0.03)	0.016

Data are presented as median (25th–75th percentile). Comparisons between smoking and non-smoking pregnant women were performed using the Mann–Whitney U test. FVC, forced vital capacity; FEV_1_, forced expiratory volume in one second; PEF, peak expiratory flow; FEF_25–75_, forced expiratory flow between 25% and 75% of FVC. Predicted values are expressed as percentages of the reference value.

**Table 3:** Respiratory muscle strength, functional exercise capacity, dyspnea, and oxygenation findings.

Variable	Non-Smokers (*n* = 55)	Smokers (*n* = 55)	*p* Value
MIP, cmH_2_O	69.6 (68.2–71.6)	64.9 (63.0–66.5)	<0.001
MIP, % predicted	96.1 (93.9–98.0)	89.5 (86.3–91.8)	<0.001
MEP, cmH_2_O	81.6 (79.2–83.3)	75.3 (73.2–76.9)	<0.001
MEP, % predicted	98.5 (96.0–100.3)	91.0 (89.6–92.7)	<0.001
6-min walk distance, m	525 (520–533)	488 (480–499)	<0.001
Baseline SmO_2_, %	72.5 (72.0–73.5)	69.9 (69.2–70.7)	<0.001
End-exercise SmO_2_, %	69.8 (69.2–70.7)	64.9 (64.3–65.6)	<0.001
ΔSmO_2_, percentage points	−2.8 (−3.8 to −1.9)	−5.0 (−6.2 to −4.4)	<0.001
Baseline Borg dyspnea score	1 (0–1)	1 (0–1)	0.684
Post-exercise Borg dyspnea score	2 (2–3)	4 (4–4)	<0.001
Baseline SpO_2_, %	96 (96–97)	96 (95–97)	0.347
Post-exercise SpO_2_, %	96 (95–96)	96 (95–97)	0.771

Data are presented as median (25th–75th percentile). Comparisons between smoking and non-smoking pregnant women were performed using the Mann–Whitney U test. MIP, maximal inspiratory pressure; MEP, maximal expiratory pressure; 6MWD, six-minute walk distance; SmO_2_, skeletal muscle oxygen saturation; SpO_2_, peripheral oxygen saturation. ΔSmO_2_ was calculated as end-exercise SmO_2_ minus baseline SmO_2_; therefore, more negative values indicate a greater exercise-related reduction in muscle oxygenation.

**Table 4:** Multiple linear regression analysis for factors associated with ΔSmO_2_.

Variable	B	Standard Error	Standardized β	*p* Value	VIF
Pack-years	−0.117	0.030	−0.512	<0.001	3.97
BMI, kg/m^2^	−0.052	0.055	−0.079	0.350	1.59
MIP (cmH_2_O)	0.121	0.071	0.242	0.093	4.63
Gestational age (weeks)	−0.105	0.037	−0.217	0.006	1.33
Hemoglobin (g/dL)	0.158	0.166	0.065	0.342	1.07

Model statistics: R^2^ = 0.541; Adjusted R^2^ = 0.519; F = 24.564; *p* < 0.001.

## Data Availability

The original contributions presented in this study are included in the article. Further inquiries can be directed to the corresponding author.
